# Caring for Offspring in a World of Cheats

**DOI:** 10.1371/journal.pbio.1001519

**Published:** 2013-03-26

**Authors:** Dieter Lukas

**Affiliations:** Department of Zoology, University of Cambridge, Cambridge, United Kingdom

## Abstract

Parents providing care to offspring face the same problem that exists in every biological system in which some individuals offer resources to others: cheaters, who exploit these benefits. In almost all species in which males contribute to parental care, females mate with multiple males. As a result, males frequently provide efforts for unrelated offspring at a cost to their own reproductive fitness. In a new study, Griffin et al. find that across a wide range of animal species, males flexibly adjust their contribution to parental care in relation to extra-pair paternity. However, adjustment is not perfect, because males are limited by the potential costs of withholding help to their own offspring, which is only outweighed if cheating occurs frequently and if providing care reduces a male's future reproductive success. These findings illustrate how in biological systems cheater and cheated can adapt to changes in each other, preventing either one from gaining control.

## Abstract 2

A comparative study of paternal care behavior shows how costs and benefits mitigate the occurrence of defense strategies against extra-pair offspring, keeping cheaters at bay but not completely out.

Whatever your personal feelings, evolutionary biologists will tell you that caring for offspring is not an easy affair. Pick up any current textbook on behavioural ecology, and you will find that the word “family” is invariably followed by the word “conflict” (e.g., [1]). Conflicts between family members arise because selection favours individuals aiming to maximize reproductive fitness, and these aims frequently collide because selection pressures differ even among related individuals [2]–[4]. Offspring can improve their reproductive fitness by obtaining the maximal amount of investment from both of their parents. However, parents frequently provide less than the maximum because any increased investment into current offspring impacts their ability to produce additional offspring in the future. Caring for offspring in all its forms is energetically expensive and may impair a parent's ability to have additional offspring in a variety of ways. For example, when a female of the golden egg bug (*Phyllomorpha laciniata*) lays her eggs on a male rather than on a plant, her offspring will have increased survival, but the father carrying the eggs has a higher risk of being eaten by a bird [5]. In bighorn sheep (*Ovis canadiensis*) [6], mothers are less likely to have a surviving offspring in the year after rearing a son, as males are generally heavier at birth and suckle more frequently because being larger provides an advantage when competing against other males. In European starlings (*Sturnus vulgaris*), males who participate less in the incubation of the offspring have a higher chance of gaining a secondary female [7].

Given the costs of providing parental care, we would expect that individuals should not expend energy if they do not gain any fitness at all, as is the case when they care for offspring that are not their own [2],[8]–[10]. Individuals that are potential victims of cheating are predicted to have evolved a range of counteradaptations to reduce the risks and costs of raising unrelated offspring [11],[12] (Table 1). Such strategies have been well documented for hosts of interspecies brood parasites, such as cuckoos or cowbirds that lay their eggs in the nests of other species who raise their young. Strategies against such parasitism include nest defence, mechanisms to recognize and expel foreign offspring, or, if parasitism cannot be avoided, adaptations to minimize the costs of caring for unrelated offspring [13].

**Table 1:** Strategies to minimize the risks and costs of being exploited by cheaters.

Strategies against cheaters	What can fathers do?	What happens in other contexts involving cheaters?
Prevent cheaters from invading	Males frequently perform mate guarding, which ensures that they sire the offspring they are going to raise [21],[22]	Bacterial species that produce common goods disperse widely and then clonally reproduce, reducing the chance of cheater encounters [35]
Recognize individual cheaters and shun them	In a few species, males appear able to recognize their own offspring, which ensures that benefits are not directed toward unrelated offspring [23]	Bird hosts of cuckoos and cowbirds produce colourful eggs, which increases their chance of recognizing the parasitic eggs [36]
Adjust contributions according to cues that indicate potential returns	Males reduce paternal care when it is likely that unrelated offspring are part of the brood, which saves energy for future attempts in which no cheaters are around (study by Griffin et al. [27])	Cleaner fish refrain from biting clients when observed by bystanders who are potential clients [37]

Cheaters, individuals who exploit the efforts of others, exist in a variety of contexts. In response, strategies have evolved that reduce the risks and costs of being cheated. The table describes three general strategies, shows how they apply to the context of fathers reducing the costs of caring for unrelated offspring including the finding by Griffin et al. [27], and provides examples from other contexts.

Cuckoldry, individuals caring for unrelated offspring, not only occurs between members of different species, but also within a species. Caring fathers are the main victims of such intraspecies cuckoldry, because high levels of sperm competition mean that males frequently have less certainty about whether they are the parent of any given offspring [14]. Despite this uncertainty, paternal care is widespread across animals because offspring are the primary way through which individuals gain reproductive fitness [15] (Figure 1). In those fish species in which parental care occurs, it is usually the male who cares for the eggs or offspring by building a nest, fanning the eggs to ensure they receive enough oxygen, or protecting offspring against predators [16]. Males of some insect [17] and amphibian species carry the eggs on their back [18]. In most bird species, females and males share the costs of building the nest, incubating the eggs, and feeding the offspring [19]. In some monogamous and social mammals, including humans, males provide food and protection for dependent offspring [20].

**Figure 1:**
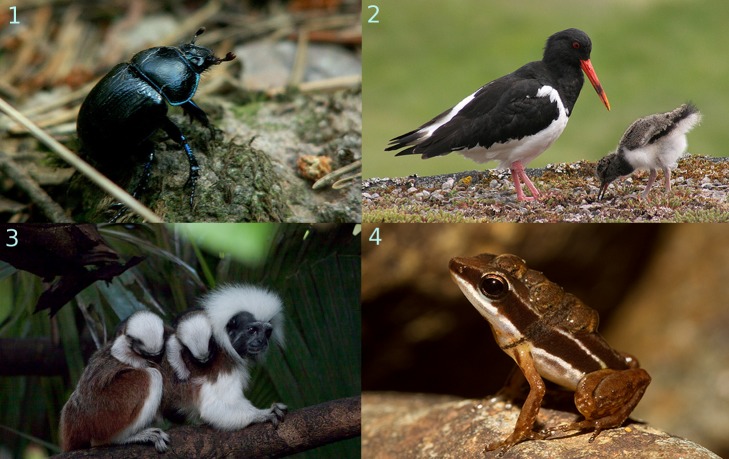
Males contribute to the raising of offspring in a variety of ways in different species. In earth-boring dung beetles (*Geotrupes vernalis*) (1) and oyster catchers (*Haematopus ostralegus*) (2), males and females live in pairs and share the burdens of providing food for their offspring. In cotton-top tamarins (*Saguinus oedipus*) (3), males carry and protect offspring as they travel with the group while they are still being nursed by their mothers. Rainforest rocket frog (*Silverstoneia flotator*) (4) mothers transfer their eggs to the male before leaving, and the father cares for the developing offspring alone. Picture credit: All pictures under Creative Commons Attribution License: (1) HaPe_Gera, http://www.flickr.com/photos/hape_gera/235786194/; (2) John Haslam, http://www.flickr.com/photos/foxypar4/511910343/; (3) Qi Wei Fong, http://www.flickr.com/photos/photo-gratis/4631252697/; (4) Brian Gratwicke, http://www.flickr.com/photos/briangratwicke/5414228931/.

There is relatively little consensus about the circumstances that explain why males do or do not adopt strategies to reduce the risks and costs of intraspecies cuckoldry. One well-documented and widespread male behaviour is mate guarding [21]; for example, mating induces rapid hormonal changes in the males of monogamous prairie voles (*Microtus ochrogaster*) that cause them to become aggressive toward conspecific strangers entering their territory and approaching the female [22]. Only a few instances of males discriminating and adjusting efforts between their own versus another male's offspring within a brood have been reported, probably because cues that directly reflect genetic relatedness are rare [23],[24]. While individuals in many species adjust their behaviour according to how closely related they are to another individual, almost all rely on cues of familiarity; for example, long-tailed tits (*Aegithalos caudatus*) learn the calls of all of the individuals they encounter during their nestling phase, and they discriminate kin based on song [25]. However, such learned “familiarity cues” do not provide a way to discriminate kin from non-kin among offspring within a clutch or brood. Rather than reduce care toward specific offspring, males might alternatively decrease their total care contribution in reproductive attempts when cues indicate that they are less likely to have sired all the offspring. Until now researchers were undecided whether and in which ecological circumstances selection acts upon males to adjust care according to their average relatedness to the offspring [10],.

In this issue of *PLOS Biology*, Griffin et al. turn to the method of phylogenetic meta-analysis to address the question of whether males show a reduction in paternal care in response to a loss of paternity [27]. Phylogenetic meta-analyses are a novel statistical approach that provide a quantitative synthesis of results across studies and across species [28],[29]. Contrary to inferences based on simple counting of the number of studies with significant results, summarizing the large number of empirical studies conducted to date in this rigorous way shows that the reduction of paternal care provided for broods that contain unrelated offspring is indeed a general biological phenomenon. Rather than being a rare behaviour that occurs under only limited circumstances, it can be found in more than 80 percent of the bird, insect, mammal, fish, and reptile species that have been studied to date. Evidence for the individual adjustment of paternal care provides an important addition to previous comparative analyses, which found that average levels of extra-pair paternity across all nests in a population covary with the average amount of care fathers provide [30],[31]. While not necessarily influenced by the same factors, differences between species ultimately derive from variation within populations, and Griffin et al.'s meta-analysis shows that variation between individual males with regard to parental effort can exist [27].

In addition, phylogenetic meta-analyses allowed Griffin et al. to detect factors that have systematic effects on the strength of the adjustment of paternal care [27]. They found that reductions in paternal care are particularly high in species that have both high rates of cheating and where investment in paternal care strongly decreases the future reproductive success of males. Adjustment of paternal care will not be selected for in species with low levels of cheating because males that withhold care would risk harming their own offspring that are part of the brood. Selection for withholding care will also be weak if the benefits of gaining additional reproductive fitness are low. This suggests that male adjustment of paternal care is not limited just by an absence of reliable cues for males to detect when they have been cuckolded, but rather it is limited if the costs of potentially harming one's own offspring outweigh the benefits of conserving energy to invest in future offspring. These findings could also inform our understanding of the evolution of interspecies cuckoldry, where it is currently unclear why individuals appear to accept parasitic cuckoo nestlings or larvae into their care in such a large number of species [13]. Based on the findings by Griffin et al. [27], future comparative studies could examine whether the frequency of cheating and the cost of caring for the stranger interact to explain the distribution of parasite acceptance.

Griffin et al.'s findings raise important new questions for the evolution of paternal care. While the presented analyses focus on males, in most of the species included in their dataset both parents contribute to the raising of offspring, and the dynamics between the sexes have important consequences on mating and care strategies [32]. A previous meta-analysis found that, in birds, females increased their parental care efforts to partially compensate for lack of care by males if males were experimentally prevented from providing for the offspring, but they also found large variation across species in female response to male reductions of care [33]. Are females in species in which males show large variation in care more likely to compensate for the loss in paternal contribution by increasing their own efforts, or does male adjustment of care affect the fitness of the current offspring? There are other possible consequences of reductions in paternal care: males could be more effective at preventing cheating during the next breeding attempt, or it could influence females to seek fewer extra-pair matings. To address these questions, long-term individual-based studies are necessary to assess how the adjustment of paternal care interacts with external conditions and other behaviours of the male and his mate.

Detailed studies are also necessary to understand why plasticity within individuals in extra-pair mating continues to exist. Given the high costs to males if females cheat, and the costs to females if males reduce their contribution to parental effort, why have both females and males not adjusted their behaviour to a stable strategy that maximizes fitness? Plastic adjustment of paternal care could be more likely in populations in which external factors lead to rapid changes in the frequency of cheating. For example, research in birds has shown that the occurrence of extra-pair paternity changes with fluctuations in the density of conspecifics within populations [34]. When drastic changes in density occur within the lifespan of males, individual responses that allow an adjustment of paternal care could be beneficial. If environmental changes influence the costs and benefits of mating strategies and the occurrence of extra-pair matings, reductions in paternal care could be the result of fathers reallocating energy to pursue extra-pair mating opportunities, rather than reducing the costs of caring for unrelated offspring.

In general, the findings by Griffin et al. are a great illustration of the evolutionary struggle inherent in any system where some individuals provide a resource that can be exploited by others. In terms of parents providing resources to offspring, these new results show that fathers in many species adjust their behaviour flexibly to prevent and punish exploiters, while minimizing the costs to both their current and future offspring [27]. Nevertheless, they might still end up caring for unrelated offspring if selection leads females to keep extra-pair matings at a level that males will tolerate. More research is needed to understand the costs and benefits of all the actors within this system: father, mothers, and their offspring, and extra-pair males and their offspring. The long-standing study of family conflict, and the variety of solutions that have been recorded in different species, offers the opportunity to generate important insights into the evolution of exploitation and the strategies that prevent it.
